# Treatment and the Pulmonary Circulation

**Published:** 1895-02-23

**Authors:** Benjamin Ward Richardson


### Treatment and the Pulmonary Circulation.

By Sir Benjamin Ward Richardson, M.D., F.R.S.

### IV.

### THE BRONCHIAL SURFACE AND SECRETION.

In the last three papers I have dealt with the general subject of "Therapeutics and the Lesser Circulation." I proceed now to a more specific exposition of the principles of treatment which should govern us in the management of the pulmonic circuit.

I. In reference to these principles of treatment we have to consider—(1) the condition of the bronchial surface in respect to the amount of secretion that is contained in it; (2) the state of the lung structure in regard to the amount of blood that is passing through it; (3) the extravasation of blood, or of the serum of the blood, into the parenchyma, or into the commencement of the bronchial system; (4) the hæmorrhagic condition of lung; (5) the exudation of fluid through the pleura; (6) the breach of the syphon action of the blood from the right to the left sides of the heart, as from paralysis of the right side, or direct obstruction due to a separation of fibrine in the right auricle, ventricle, or pulmonary artery.

I do not think there is ever a condition of the body in which pyrexia forms one of the symptoms of disease without the occurrence of some divergence from health in the state of the bronchial mucous membrane and its secretion. As a general rule, in all cases of pyrexia where the skin is dry there is dryness also of the mucous membrane of the bronchial surface. The use of the stethoscope has for so many years been a part of my work, I have scarcely ever treated a febrile case of any kind without an examination of the lung, and I can recall no instance in which with pyrexia there has not been some sign of dryness of the bronchial surface in the first stage of disease, whatever that may be. I have noticed in the simple febrile state which is called commonly "surgical pyrexia," the elevation of temperature following upon a surgical operation, which Hunter thought was a natural and even useful reaction, that there is a slight indication of arrest of bronchial secretion. The patient may not be aware of any difficulty in breathing, or of any sign indicating pulmonary affection, but the stethoscope reveals that the natural vesicular murmur is modified by the absence of the ordinary quantity of bronchial secretion. There is nothing surprising in this variation; no more reason for surprise than exists in the dryness of the skin; because the membrane itself may be considered as a continuation of a modified cutaneous surface over which a current of air, or of gases, is constantly passing and repassing. These currents of air may practically be the same in regard to the current that is inspired, but the expired current must naturally vary in its temperature according to the variations of the blood that is brought up from the body to the lungs, and from which the expiratory current of carbonic acid, water vapour, nitrogen, and oxygen is derived.

Ordinarily there is a distinct condensation of water derived from the air in ordinary inspiration, unless the air be unusually dry. If the air be saturated with moisture there is always condensation; if it be half saturated there is some condensation on the bronchial surface; if it be deprived of moisture altogether there may still be moisture, in which case the fluid condensed is derived from the blood, as if there were what might be termed a watery hæmorrhage into the minuter bronchial tubes. If the temperature of the body be very high, and if at the same time the air inspired be charged with moisture, there may still be dryness of the surface; the warmth of the gases exhaled from the blood being sufficient to cause dissipation of the water inspired. These are all important points in practice, though minor in detail; but that which is most important is the condensation in the finer portions of the bronchial tubes at times when the action of the heart is feeble, when the circulation is low, and when the air is charged with moisture, and is low in degree. Under such circumstances there is a double source of condensation, and there is produced the condition which I have elsewhere named *hydrops bronchialis*—dropsy of the bronchial system. When the mind is educated to detect this phenomenon, it becomes astonished at witnessing what an immense number of deaths occur from the one cause. Amongst aged people who die rather suddenly from bronchial disease—chronic bronchitis, as it is often designated—hydrops bronchialis is the most common cause of death. The lungs have lost much of their resiliency, the blood circulation over them is feeble, the temperature of the blood is below the natural standard, the bronchial surfaces are dilated, and the train is laid for accumulation of fluid by condensation beginning in the minute bronchial tubes. In such persons, though they may seem to be in fair average health, and may be going about performing their part of the ordinary routine of life, become exposed—accidentally or thoughtlessly—to a cold damp air, or to some deprivation in food that brings down their circulation, there commences a slightly reduced temperature of the blood, and just as water condenses at the dew point, some trifling condensation of water commences in their bronchial periphery. The condensation once started proceeds; there is further separation of the blood from the air; oxidation is coincidentally reduced, and decline of temperature from two causes of obstruction begins, and progresses. In time faint bronchial rale is detected by the stethoscope, and we say then that bronchitis of a chronic character is presented. Under favouring circumstances the heart may regain power; evaporation from the pulmonary organs may return, and the resistance in the pulmonary circuit may be removed, with recovery. In other instances the condensation under the twofold difficulty may continue, with the result of death from drowning by the bronchial fluid of the body itself. Amongst enfeebled people who are free from special organic diseases this is the cause of death, probably in 90 per cent. of instances, and might be classified as natural euthanasia.

